# Supplementary material for: Lanatoside C Inhibits Proliferation and Induces Apoptosis in Human Prostate Cancer Cells Through the TNF/IL-17 Signaling Pathway
Source: Int J Mol Sci. 2025 Mar 12;26(6):2558. doi: 10.3390/ijms26062558 (PMC11941998; doi:10.3390/ijms26062558)
Supplement: Supplementary file 1 [file ijms-26-02558-s001.zip › ijms-3475369-supplementary.pdf]

Supplementary data

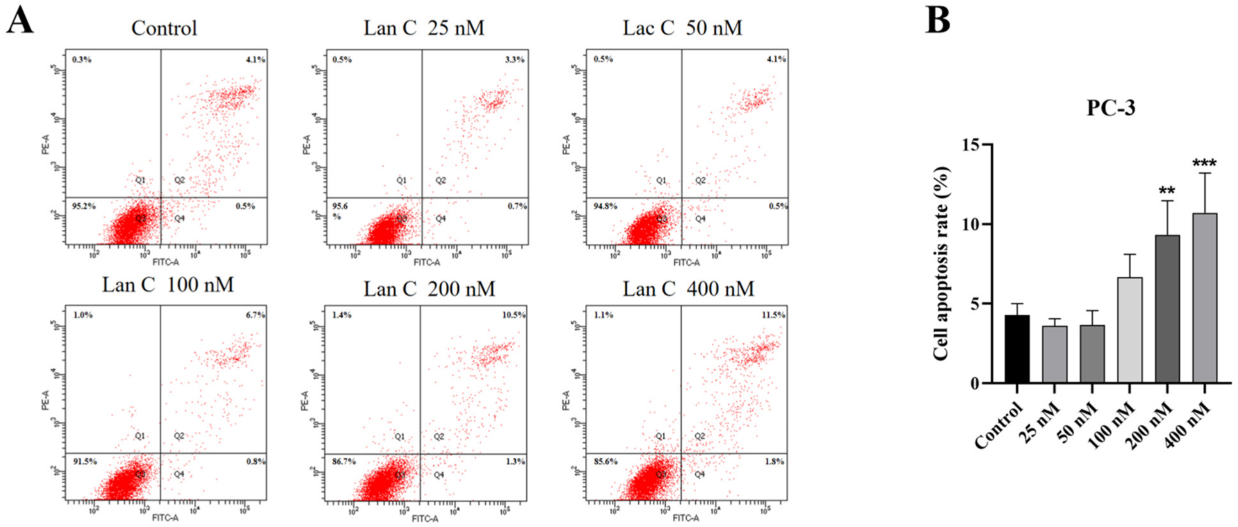

Figure S1. Apoptotic scatter plots (A) and statistical histogram (B) of apoptotic proportions of human prostate cancer cells treated with PC-3 by Lan C for 48 h. Data are presented as the mean ± SD of three independent experiments. Statistical analysis was performed after normality testing, using ANOVA for between-group comparisons. If homo-geneity of variance was assumed, the LSD test was used; if the variance was heterogeneous, Dun-nett's post hoc test was applied. Comparison with the control group: ** $p < 0.01$, *** $p < 0.001$.

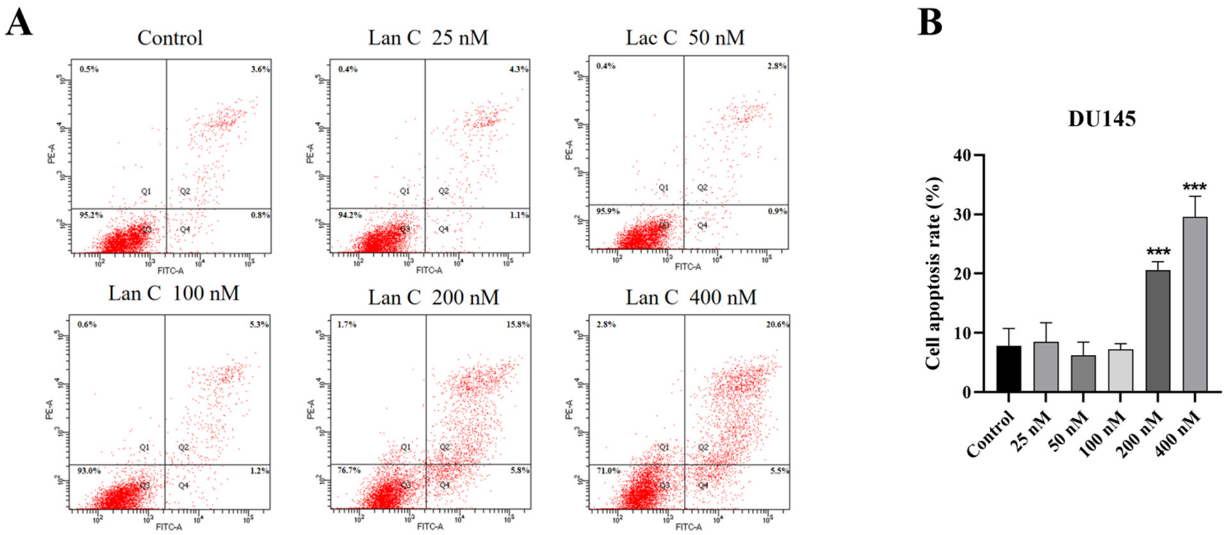

Figure S2. The scatter plot (A) of apoptosis and the statistical histogram (B) of the proportion of apoptosis in each group after treatment of human prostate cancer cells DU145 by Lan C for 48 h. Data are presented as the mean ± SD of three independent experiments. Statistical analysis was performed after normality testing, using ANOVA for between-group comparisons. If homo-geneity of variance was assumed, the LSD test was used; if the variance was heterogeneous, Dun-nett's post hoc test was applied. Comparison with the control group: *** $p < 0.001$.

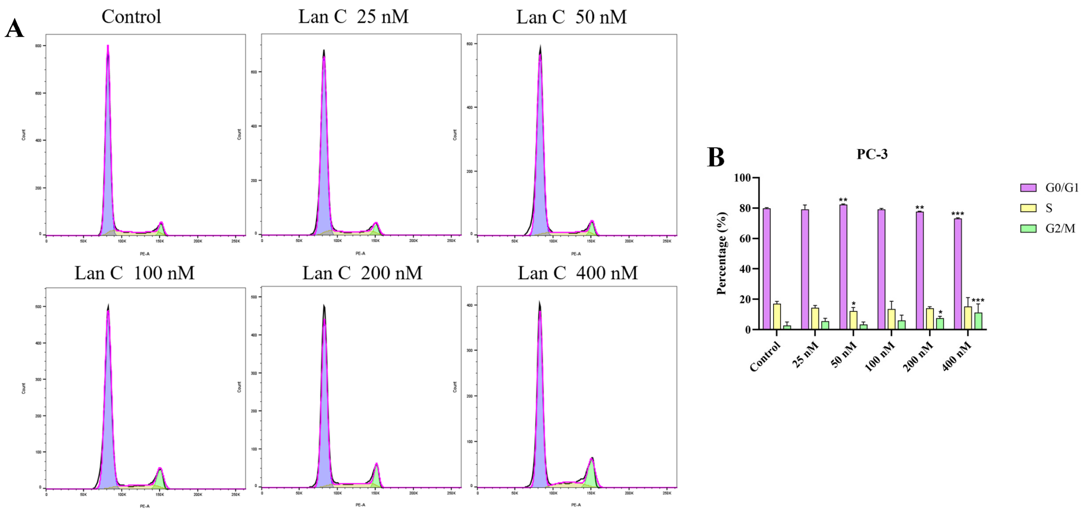

Figure S3. Cell cycle distribution diagram (A) and statistical analysis diagram (B) of human prostate cancer cells treated with PC-3 by Lan C for 48 h. Data are presented as the mean ± SD of three independent experiments. Statistical analysis was performed after normality testing, using ANOVA for between-group comparisons. If homo-geneity of variance was assumed, the LSD test was used; if the variance was heterogeneous, Dun-nett's post hoc test was applied. Comparison with the control group: * $p < 0.05$, ** $p < 0.01$, *** $p < 0.001$.

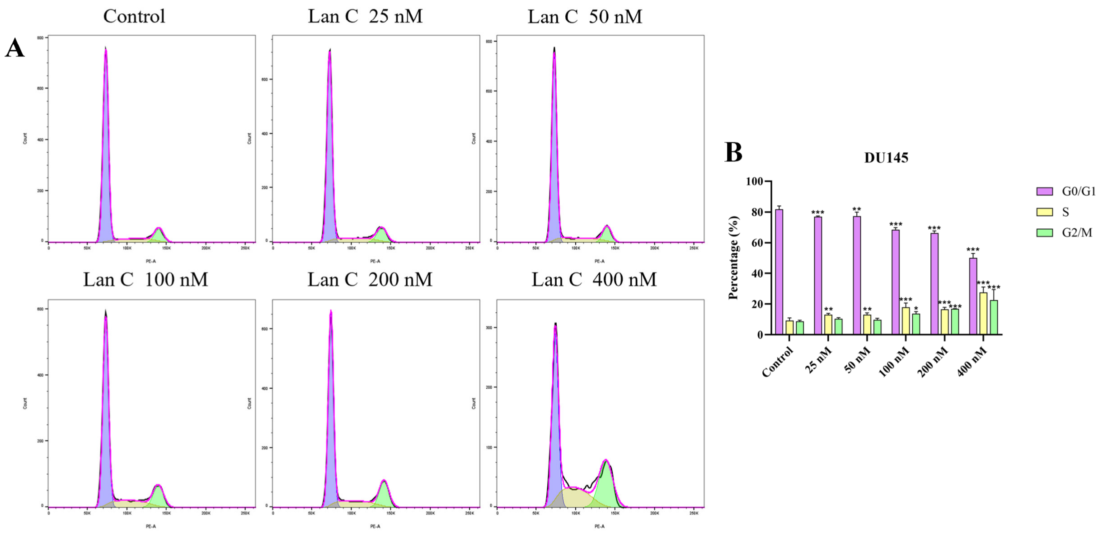

Figure S4. Cell cycle distribution diagram (A) and statistical analysis diagram (B) of human prostate cancer cells DU145 treated by Lan C for 48 h. Data are presented as the mean ± SD of three independent experiments. Statistical analysis was performed after normality testing, using ANOVA for between-group comparisons. If homo-geneity of variance was assumed, the LSD test was used; if the variance was heterogeneous, Dun-nett's post hoc test was applied. Comparison with the control group: * $p < 0.05$, ** $p < 0.01$, *** $p < 0.001$.

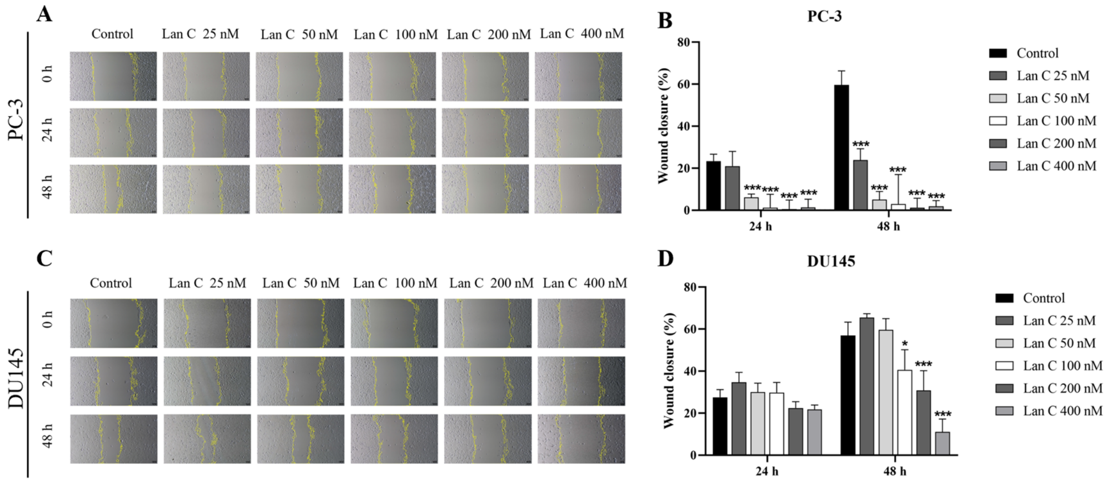

Figure S5. Lan C inhibits lateral migration of human prostate cancer cells. (A, B) Scratch wound healing assays were performed to assess the migration ability of PC-3 prostate cancer cells after treatment with 25-400 nM Lan C for 24 h and 48 h (40×, scale bar = 100 μm). (C, D) Scratch wound healing assays were performed to assess the migration ability of DU145 prostate cancer cells after treatment with 25-400 nM Lan C for 24 h and 48 h (40×, scale bar = 100 μm). Data are presented as the mean ± SD of three independent experiments. Statistical analysis was performed after normality testing, using ANOVA for between-group comparisons. If homogeneity of variance was assumed, the LSD test was used; if variance was heterogeneous, Dunnett's post-hoc test was applied. Comparison with the control group: $^{*}p < 0.05$, $^{**}p < 0.01$, $^{***}p < 0.001$.

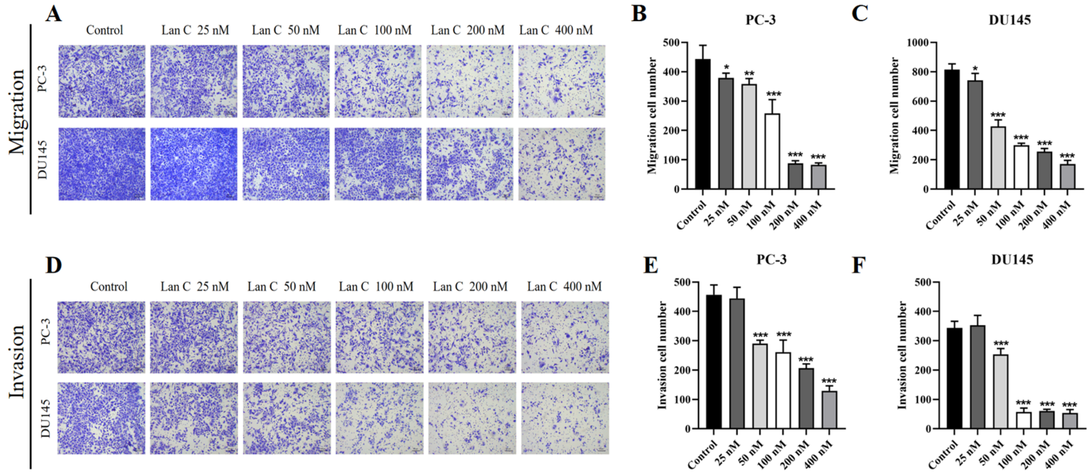

Figure S6. Lan C inhibits the longitudinal migration and invasion of human prostate cancer cells. (A, B, C) The effect of 25-400 nM Lan C on the longitudinal migration of human prostate cancer cells PC-3 and DU145 after treatment for 48 h (40×, scale bar = 100 μm). (D, E, F) The effect of 25-400 nM Lan C on the invasion ability of human prostate cancer cells PC-3 and DU145 after treatment treatment for 48 h (40×, scale bar = 100 μm). Data are presented as the mean ± SD of three independent experiments. Statistical analysis was performed after normality testing, using ANOVA for between-group comparisons. If homogeneity of variance was assumed, the LSD test was used; if variance was heterogeneous, Dunnett's post-hoc test was applied. Comparison with the control group: $^{*}p < 0.05$, $^{**}p < 0.01$, $^{***}p < 0.001$.
